# Influence of obesity on the outcome of non-surgical periodontal therapy - a systematic review

**DOI:** 10.1186/s12903-016-0272-2

**Published:** 2016-09-02

**Authors:** Fabienne A. Gerber, Philipp Sahrmann, Oliver A. Schmidlin, Christian Heumann, Jürg Hans Beer, Patrick R. Schmidlin

**Affiliations:** 1 Clinic of Preventive Dentistry, Periodontology and Cariology, Center of Dental Medicine, University of Zurich, Plattenstrasse 11, CH-8032 Zurich, Switzerland; 2 Department of Internal Medicine, Cantonal Hospital Baden, Baden, Switzerland; 3 Department of Statistics, Ludwig-Maximilians-University of Munich, Munich, Germany

**Keywords:** Obesity, Chronic periodontitis, Non-surgical periodontal therapy, Outcome

## Abstract

**Background:**

Obesity and periodontitis are important chronic health problems. Obesity is associated with an increased prevalence of periodontitis. Whether obesity also affects the outcome of non-surgical periodontal therapy is to date still unclear.

**Methods:**

A systematic review of studies referenced in SCOPUS, MEDLINE, PubMed, Cochrane, CINAHL, Biosis and Web of Science was performed. Titles, abstracts and finally full texts were scrutinized for possible inclusion by two independent investigators. Quality and heterogeneity of the studies were assessed and the study designs were examined. Probing pocket depth reduction was analyzed as primary surrogate parameter for therapeutic success after non-surgical periodontal therapy.

**Results:**

One-hundred-and-fifty-nine potentially qualifying studies were screened. Eight studies fulfilled the inclusion criteria and were analyzed. Three of eight studies failed to show an influence of obesity on pocket depth reduction after non-surgical therapy. The remaining five studies documented a clear negative effect on the outcome of non-surgical periodontal therapy. The finally included studies did not correspond to the highest level of quality (RCTs). Due to the heterogeneity of the data a meta-analysis was not possible.

**Conclusion:**

The literature on the effect of obesity on the treatment outcome of non-surgical periodontal therapy remains controversial. The data, however, support that obesity is not only a factor associated with poorer periodontal health but might also result in inferior response to non-surgical treatment of periodontitis.

## Background

The prevalence of obesity is increasing worldwide and is becoming one of the most important health hazards [1], as obesity is highly associated with increased overall morbidity and mortality [2].

Obesity is defined with a body mass index (BMI; body weight in kilogram divided by the square of the height in meters (kg/m^2^)) of at least 30.0 kg/m^2^ [3], whereas overweight is defined with a BMI of 25–29.9 kg/m^2^. Normal weight is characterized by a BMI ranging between 19 to 24.9 kg/m^2^ [4]. In this context, BMI seems a valuable parameter to predict obesity-related disease risks in a wide range of populations [2]. There are, however, some limitations: Firstly, risk assessment by BMI is less applicable in persons over 65 years of age because they generally have a higher body fat content for the same BMI. Secondly, the abdominal (central, visceral, android) type of obesity, which is more often seen in men, is associated with higher morbidity than the rather female type of gluteofemoral (peripheral, gynoid) obesity and, thirdly, the BMI cut-off points for overweight and obesity are too high for Asian people [2]. In addition, current large studies have indicated that measurement of waist circumference (WC) or waist-to-hip-ratio (WHR) may be a better disease risk predictor than BMI [5, 6]. There is, however, currently intensive research and debate as to whether BMI, WC, WHR, or all of them should be used to assess disease risk [2].

For the purpose of this systematic review, however, only BMI is the most frequently reported data of obesity in a large number of studies.

Adipose tissue contains usually 5-10 % macrophages, but the adipose tissue of obese patients shows up to 60 % macrophage infiltration [4]. Adipocytes secrete bioactive molecules called adipokines, that can modify or trigger inflammation and fat metabolism locally or systemically as signaling molecules to liver, muscle and endothelium [4]. Therefore, the adipose tissue can be considered as an important metabolically active endocrine organ [4].

This explains how obesity acts as a risk factor for several chronic diseases: Hypertension, type 2 diabetes, dyslipidemia, and coronary heart disease are so closely related to obesity that obesity itself is often considered to be a systemic disease. This disease also affects dental health [7]. Accordingly obese persons require attention of physicians and dentists [2].

Among dental pathologies, periodontitis is a very common, primarily bacterial inflammatory disease, which destroys teeth surrounding soft tissues and bone. It leads to pocket formation and ultimately to loss of teeth if no effective treatment is applied [8]. Periodontitis is no longer considered only an oral health issue but also a public health problem, as it constitutes a risk factor for cardiovascular conditions, poor glycemic control in diabetics and adverse outcome of pregnancy [4, 8]. These correlations coincide with obesity and general health.

Recently, it has been suggested that obesity is a possible risk factor for periodontitis [8]. One study identified obesity even as the second strongest risk factor for periodontitis preceded only by smoking [9]. The first report on the relationship between obesity and periodontal disease appeared in 1977. Perlstein and co-workers [10] found greater alveolar bone resorption in obese than in non-obese rats. Under healthy oral conditions, obesity itself did not promote periodontal damage, but in the presence of bacterial plaque accumulation periodontal inflammation was more severe in obese than in non-obese animals. With concomitant arterial hypertension, plaque accumulation caused even more pronounced periodontal destruction than with obesity alone. These results suggest that a combination of risk factors, such as the one defined by the metabolic syndrome, elicit a more severe periodontal effect [10, 11]. Chaffee et al. [12] found in their meta-analysis an increased prevalence odds ratio for obesity among subjects with periodontal disease of approximately one-third, a greater mean clinical attachment loss (CAL) among obese individuals, a higher BMI among subjects with periodontal disease, and a trend for linear increase in the odds of periodontal disease with increasing BMI [4, 12]. Finally the association reported between obesity and periodontitis was less strong than that reported between periodontal disease and adverse pregnancy outcomes [12, 13] or cardiovascular events [12, 14]. There seems, however, to be a stronger obesity-periodontitis association in women, non-smokers and younger individuals than in the general adult populations [12]. In addition, smoking remains another well-studied predisposing factor for periodontitis [12, 15, 16]. Thus, BMI and smoking share a complex relationship [17]. This relationship can be inverse in certain populations [12, 18, 19].

The biological mechanism by which obesity predisposes to periodontitis is not fully understood [8]. Compared to individuals with normal weight individuals with obesity have higher levels of circulating tumor necrosis factor-α (TNF-α) and interleukin-6 (IL-6), which are also secreted from adipose tissue and are involved in the pathophysiology of both obesity and periodontitis. Not surprisingly, serum levels of these cytokines decrease with loss of weight [20].

The objective of this systematic review was to study the hypothesis whether the clinical outcome, in terms of pocket depth reduction, after non-surgical periodontal therapy in non-obese is better than in obese individuals. To verify this hypothesis, we systematically reviewed all retrievable, qualitatively adequate clinical investigations, which focused on this topic.

## Methods

The review was conducted according to the PRISMA criteria [21]. The research question was explored using the *PICO* method [22). The focused question addressed was:

Does non-surgical periodontal therapy (I) have a different outcome in obese chronic periodontitis patients (P), than in non-obese chronic periodontitis patients (C), regarding periodontal pocket depth reduction as the main clinical periodontal parameter (O).

### Search strategy and review process

An electronic search of SCOPUS, MEDLINE, PubMed, Cochrane, CINAHL, Biosis and Web of Science was carried out considering articles published up to January 2016 in English or German language. The search was performed in two steps. The first electronic search started at 20.11.14 and an update has been done at 5.1.16.

This is shown in Table 1. For the database search, a combination of subject headings (MeSH terms and CINAHL headings) and free text search was used. An example of a detailed strategy (Medline/OvidSP) is shown in Table 2.

**Table 1:** Search protocols with the respective number of references dated from November 11 2014 (1) and January 1 2016 (2)

	References 1 & 2	References after deduplication 1 & 2
Scopus	40 + 14	9 + 5
Medline	42 + 8	39 + 7
PubMed	6 + 11	2 + 11
Cochrane	12 + 2	2 + 1
CINAHL	48 + 15	42 + 13
Biosis	25 + 7	10 + 1
Web of Science	38 + 13	16 + 1
Pool	211 + 70	120 + 39

After deduplication 159 references

**Table 2:** Medline search strategy

Step	Query	Hits
1	Exp Periodontitis/AND (adult OR chronic*).ti,ab.	5703
2	Chronic periodontitis/OR periodontal pocket/	7004
3	2 OR 3	10454
4	((adult OR chronic*) adj3 periodontiti*).ti,ab.	5352
5	(attachment adj3 loss).ti,ab. AND ((clinical OR periodontal) adj3 attachment).ti,ab.	1305
6	(periodontal adj3 pocket).ti,ab.	1066
7	OR/3-7	12910
8	Dental scaling/ OR “root planing”/	3479
9	((subgingival OR root OR supragingival) adj3 (scaling* OR planing*)).ti,ab.	2288
10	(deep adj3 (scaling* OR cleaning)).ti,ab.	62
11	((conventional OR inital OR “non surgical” OR non-surgical OR nonsurgical) adj3 (treatment OR therapy)).ti,ab.	33417
12	OR/8-11	37131
13	7 AND 12	2184
14	Exp Obesity/	154540
15	Exp Body Weight/	369850
16	(overweight OR obese OR obesity OR adiposit* OR BMI OR “body mass index”).ti,ab.	300847
17	(cardiolipin OR lipid* OR leptin).ti,ab.	395517
18	“waist to hip”.ti,ab.	9048
19	(body adj3 weight).ti,ab.	163760
20	OR/14-19	954233
21	13 AND 20	42

The asterisk represents a wildcard and can be used in all search fields that allow words and phrases

The same search protocol was applied to all databases.

Two of the authors (FAG, PRS) screened the titles for potential eligibility according to the inclusion criteria. Based on the abstract screening, 18 studies were selected for full text review. Scores were independently allocated by both authors to each publication according to their suitability for the present review (see inclusion criteria). Any discrepancies were resolved by consensus.

### Inclusion criteria

To be included studies had to be clinical interventional studies regarding the outcome of non-surgical periodontal therapy in obese or non-obese patients. The studies had to display the diagnosis of chronic periodontitis. Key parameters to be reported were data for pocket probing depth (PPD) and BMI.

### Exclusion criteria

Studies were excluded for the following reasons: animal studies, case reports, commentaries, unsuitable exposure or outcome measures, confounding medical diagnoses (e.g. pregnancy or any systemic disease, such as diabetes, in addition to metabolic syndrome), confounding *systemic* medical treatments such as immunosuppressive treatments, cortisone or antibiotic treatment as well as confounding *local* treatments such as treatment of peri-mucositis, gingival overgrowth or surgical periodontal treatment. Studies including either the diagnosis of aggressive periodontitis or of peri-implantitis were excluded as well.

### Outcome measures

The primary outcome measure is PPD after non-surgical periodontal treatment.

### Data extraction

A list with exclusion reasons for each paper was generated. Total number of patients, demographic data, origin of study, outcome measurements 2, 3, 6 and 12 months after therapy and the impact of obesity on the treatment-outcome were extracted. In addition, the exact definition of chronic periodontitis, the assessment of the periodontal disease and the number of smokers included in the studies were summarized. Data on the individual definition of obesity and the systemic examinations were also collected. Non-surgical periodontal treatment measures, treatment time, periodontal maintenance and adverse events were also recorded for each study separately (Tables 3, 4, 5, 6 and 7).

**Table 3:** Excluded studies

Excluded studies	Reason for exclusion
Europerio 4 2003 [37)	Not addressing research question
Abstracts for the Royal Australian and New Zealand College of Psychiatrists 2005 [38]	Not addressing research question
5th Joint Meeting of the European Tissue Repair Society and the Wound Healing Society 2009 [39]	Not addressing research question
Recently published abstracts [40]	Not addressing research question
DENTSPLY Posters 2012 [41]	Not addressing research question
HealthBeat 2007 [42]	Not addressing research question
Abstracts for Poster Presentations 2011 [43]	Not addressing research question
Abstracts for the International Symposium on Dental Hygiene 2013 [44]	Not addressing research question
Abou Sulaiman et al. 2010 [45]	Not addressing research question
Acharya et al. 2010 [46]	No PPD reduction data
Akpinar et al. 2013 [47]	Not addressing research question
Altay et al. 2013 [20]	No PPD reduction data
Armstrong et al. 2010 [48]	Not addressing research question
Arora et al. 2013 [49]	Not addressing research question
Basegmez et al. 2011 [50]	Not addressing research question
Bresolin et al. 2013 [51]	Not addressing research question
Bresolin et al. 2014 [52]	Not addressing research question
Caspersen et al. 2012 [53]	Not addressing research question
Caula et al. 2014 [54]	No comparison
*Caula* et al. 2014 [54]	Duplicate
Chapple et a. 2006 [55]	Not addressing research question
Chapple et al. 2007 [56]	Not addressing research question
Chandni et al. 2015 [57]	Not addressing research question
Chaston et al. 2014 [58]	Not addressing research question
Chee et al. 2008 [59]	Inclusion of diabetes
Chee et al. 2013 [60]	Missed intervention, inclusion of diabetes
Chen et al. 2012 [61]	Inclusion of diabetes
D’Aiuto et al. 2004 [62]	Not addressing research question
D’Aiuto et al. 2004 [63]	Not addressing research question
D’Aiuto et al. 2005 [23]	No comparison
D’Aiuto et al. 2006 [64]	Not addressing research question
Deppe et al. 2010 [65]	Not addressing research question
Dodington et al. 2015 [66]	No obesity data
Draper et al. 2010 [67]	Not addressing research question
Duan et al. 2009 [24]	Written in Chinese
Edwards et al. 2006 [68]	Not addressing research question
Efurd et al. 2012 [69]	Not addressing research question
Elliott-Smith et al. 2011 [70]	Not addressing research question
Engebretson et al. 2014 [71]	Not addressing research question
Fairfield et al. 2010 [72]	Not addressing research question
Fang et al. 2015 [73]	Inclusion of end-stage renal disease patients
Fentoglu et al. 2010 [74]	Statin intake
Fine et al. 2007 [75]	Not addressing research question
Fokkema et al. 2012 [76]	Not addressing research question
Fu et al. 2015 [77]	No comparison
Garcia et al. 2010 [78]	Not addressing research question
Giblin et al. 2014 [79]	Inclusion of prediabetes
Giblin et al. 2014 [79]	Duplicate
Gluch et al. 2007 [80]	Inclusion of diabetes
Goldie et al. 2002 [81]	Not addressing research question
Goldie et al. 2004 [82]	Not addressing research question
Goldie et al. 2005 [83)	Not addressing research question
Griffin et al. 2012 [84]	Not addressing research question
Gurenlian et al. 2006 [85]	Not addressing research question
Gurenlian et al. 2009 [86]	Not addressing research question
Hammaker et al. 2010 [87]	Not addressing research question
Horwitz et al. 2007 [88]	Not addressing research question
Hovliaras-Delozier et al. 2008 [89]	Not addressing research question
Ide et al. 2004 [90]	No obesity data
Ide et al. 2007 [91]	Not addressing research question
Iwamoto et al. 2003 [25]	Application of antibiotics
Jahn et al. 2004 [92]	Not addressing research question
Jahn et al. 2015 [93]	Not addressing research question
Jaiswal et al. 2015 [94]	No treatment (SRP)
Janket et al. 2014 [95]	Meta-analysis
Janket et al. 2014 [95]	Duplicate
Jared et al. 2008 [96]	Not addressing research question
Jared et al. 2008 [96]	Duplicate
Jiang et al. 2013 [97]	Not addressing research question
Kamil et al. 2011 [98]	No obesity data
Kamilov et al. 1998 [99]	Not addressing research question
Kapellas et al. 2013 [100]	No obesity data
Kardeşler et al. 2010 [26]	Inclusion of diabetes
Keller et al. 2015 [101]	Systematic review
Kiany et al. 2014 [102]	No obesity data
Kiany et al. 2015 [103]	Duplicate
Kipp et al. 2003 [104]	Not addressing research question
Kudva et al. 2010 [105]	Inclusion of diabetes
Kumar et al. 2015 [106]	Inclusion of diabetes
Kurti et al. 2007 [107]	Not addressing research question
Kurtis et al. 2007 [108]	Duplicate
Lee et al. 2014 [109]	Not addressing research question
Li et al. 2013 [110]	No obesity data
Ling et al. 2012 [111]	Inclusion of diabetes
Lo et al. 2011 [112]	Not addressing research question
Malhotra et al. 2012 [113]	No obesity data
Mancl et al. 2013 [114]	Not addressing research question
Martinez et al. 2014 [115]	Supplementation of ω-3 eicosapetaenoic acid
Martinez et al. 2014 [116]	One year of omega-3 supplementation
Matlock et al. 2012 [117]	No treatment (SRP)
McDaniel et al. 2013 [118]	Not addressing research question
Meharwade et al. 2014 [119]	Local drug delivery
Merchant et al. 2014 [120]	Inclusion of diabetes
Michalowicz et al. 2014 [121]	Inclusion of diabetes
Mizrak et al. 2006 [122]	Not addressing research question
Moeintaghavi et al. 2012 [123]	Inclusion of diabetes
Moravec et al. 2011 [124]	No treatment (SRP)
Muthu et al. 2015 [125]	No obesity data
Nassar et al. 2012 [126]	Inclusion of diabetes
Newton et al. 2011 [127]	Not addressing research question
Nichols et al. 2001 [128]	Not addressing research question
Nielsen et al. 2000 [129]	Not addressing research question
Novakovic et al. 2013 [130]	Not addressing research question
Novakovic et al. 2014 [131]	Not addressing research question
Oliveira et al. 2011 [132]	Not addressing research question
Olsen et al. 2006 [133]	Not addressing research question
Paquette et al. 2008 [134]	Not addressing research question
Perayil et al. 2014 [135]	No obesity data
Perayil et al. 2014 [136]	Duplicate
Phillips et al. 2013 [137]	Not addressing research question
Pradeep et al. 2007 [138]	Not addressing research question
Price et al. 2010 [139]	Not addressing research question
Qiqiang et al. 2012 [140]	No obesity data
Radafshar et al. 2012 [141]	Not addressing research question
Radnai et al. 2009 [142]	Not addressing research question
Raghavendra et al. 2012 [143]	Not addressing research question
Ramirez et al. 2011 [144]	No obesity data
Rasch et al. 1995 [145]	Not addressing research question
Sadatmansouri et al. 2006 [146]	Not addressing research question
Saffi et al. 2013 [147]	Inclusion of patients with coronary disease
Saffi et al. 2013 [147]	Duplicate
Saffi et al. 2014 [148]	Inclusion of patients with coronary artery disease
Shruti et al. 2010 [27]	No obesity data
Sembene et al. 2000 [149]	Not addressing research question
Sengupta et al. 1990 [150]	Not addressing research question
Sgolastra et al. 2013 [36]	Meta-Analysis
Shimada et al. 2010 [151]	No comparison
Shimoe et al. 2011 [152]	Case report
Singh et al. 2014 [153]	Not addressing research question
Siqueira et al. 2013 [154]	Not addressing research question
Stewart et al. 2001 [155]	Inclusion of diabetes
Talbert et al. 2006 [156]	Inclusion of diabetes
Tamaki et al. 2009 [157]	No obesity data
Tamaki et al. 2011 [158]	No obesity data
Tawfig et al. 2015 [159]	No comparison
Teles et al. 2012 [160]	No comparison
Toker et al. 2012 [161]	Not addressing research question
Tuter et al. 2007 [162]	Inclusion of patients with systemic disease
Vardar et al. 2003 [163]	Not addressing research question
Van Dyke et al. 2015 [164]	Not addressing research question
Vyas et al. 2000 [165]	Not addressing research question
Wahid et al. 2013 [166]	Not addressing research question
Wang et al. 2013 [167]	Inclusion of aggressive periodontitis
Wehmeyer et al. 2013 [168]	Not addressing research question
Wei et al. 2010 [169]	No obesity data
Williams et al. 2007 [148]	No obesity data
Williams et al. 2009 [170]	Not addressing research question
Williams et al. 2009 (171]	Not addressing research question
Wood et al. 2006 [172)	Not addressing research question
Wu et al. 2015 [173)	Not addressing research question
Zare et al. 2014 [174)	Not addressing research question
Zhou et al. 2013 [175]	Inclusion of patients with systemic disease
Zuza et al. 2011 [176)	No PPD reduction data

**Table 4:** Probing pocket depth (PPD [mm]) as primary outcome variable

Author & year	Total number of patients Age (mean ± SD) Country	Outcome after 2 months mean (± SD) at baseline – mean (± SD) at reassessment	Outcome after 3 months mean (± SD) at baseline – mean (± SD) at reassessment	Outcome after 6 months mean (± SD)at baseline – mean (± SD) at reassessment	Outcome after 12 months mean (± SD)at baseline – mean (± SD) at reassessment	Impact of obesity on the treatment - outcome mean – reduction of PPD (± SD)		
Al – Zahrani et al. 2012 [8]	Total number of patients: 40 (only women) Normal-weight: 20 Obese: 20 Age: Normal-weight: 43.4 (±7.8) Obese: 44 (±8.4) Country: Saudi Arabia	Total sample: 2.65 (±0.47) – 2.44 (±0.41) *p*-value = 0.001	n.r.	n.r.	n.r.	Normal-weight: 0.19 (±0.27) Obese: 0.23 (±0.33) *p*-value = 0.663 (n. sig.)		
Bouaziz et al. 2015 [28]	Total number of patients: 36 Normal-weight: 18 Obese: 18 Age: Range from 22 to 78 Normal-weight: 51.00 (±13.1) Obese: 51.3 (±16.7) p-value: n. sig. Country: France	n.r.	Normal-weight: 3.33 (±0.92) – 2.71 (±0.40) *p*-value: n. sig. Obese: 3.22 (±0.76) – 2.78 (±0.55) *p*-value: n. sig.	Normal-weight: 3.33 (±0.92) 2.45 (±0.53) *p*-value < 0.05 Obese: 3.22 (±0.76) – 2.43 (±0.49) *p*-value < 0.05	n.r.	Summary of Multivariable Analysis Results (Coefficient, 95 % Cl): △ %PD > 5mm after 3 months to obese (Model 2): *p*-value = 0.02 △ %PD > 5mm after 6 months to obese (Model 2): *p*-value = 0.005 Nb △PD > 2mm after 3 months to Obese (Model 2); *p*-value = 0.02 Nb △PD > 2mm after 6 months to Obese (Model 2); *p*-value = 0.06 (n.sig.)		
Duzagac et al. 2015 [29]	Total number of patients: 45 Normal weight patients with periodontitis (CPNW): 15 Obese patients with periodontitis (CPO): 15 Normal weight periodontally healthy control subjects (PHNW): 15 Age: Range from 28 to 52 CPNW: 41.06 (±7.10) CPO: 40.66 (±7.98) PHNW: 39.66 (±6.53) *p*-value: n. sig. Country: Turkey	n.r.	CPNW: 3.19 (±0.38) –2.51 – (±0.06) *p*-value = 0.001 CPO: 4.10 (±0.50) – 2.55 (±0.45) *p*-value = 0.001	n.r.	n.r.	Number of sites with PD < 4 mm: Baseline: CPO: 1706 CPNW: 1766 3 months: CPO: 2214 CPNW: 2206 % of sites with PD < 4 mm: Baseline: CPO: 75.4 CPNW: 77.9 3 months: CPO: 97.9 CPNW: 97.3 Similar clinical healing; (n. sig.)	Number of sites with PD ≥ 4 mm: Baseline: CPO: 556 CPNW: 502 3 months: CPO: 48 CPNW: 32 % of sites with PD ≥ 4 mm: Baseline: CPO: 48 CPNW: 22.1 3 months: CPO: 2.1 CPNW: 2.7 Similar clinical healing; (n. sig.)	
Eldin et al. 2013 [30]	Total number of patients: 26 Overweight: 12 Obese: 14 Age: Range from 35 to 48 Country: Egypt	n.r.	Overweight: 0.6 (±1.29) – 0.75 (±2.12) *p*-value: n.r. Obese: 3.15 (±2.43) – 1.59 (±2.34) *p*-value: n.r.	n.r.	n.r.	Overweight: 0.784 Obese: 0.209 *p*-value = 0.326 (n. sig.)		
Gonçalves et al. 2015 [31]	Total number of patients: 39 Without obesity: 21 With obesity: 18 Age: Without obesity: 48.4(±9.5) With obesity: 48.8 (±5.9) p-value= 0.85 (n. sig.) Country: Brazil	n.r.	Without obesity: 3.4 (±0.6) – 3.0 (±0.5) *p*-value: sig. With obesity: 3.6 (±0.6) – 3.3 (±0.6) *p*-value: sig.	Without obesity: 3.4 (±0.6)- 2.7 (±0.6) *p*-value: sig. With obesity: 3.6 (±0.6) – 3.0 (±0.5) *p*-value: sig.	n.r.	Without obesity Full-mouth sites: 0-3 months: 0.44 (±0.06) 0-6 months: 0.73 (±0.07) Initially moderate sites (PD 4-6mm): 0-3 months: 0.87 (±0.12) 0-6 months: 1.38 (±0.14) Initially deep sites (PD ≥ 7mm): 0-3 months: 1.86 (±0.25) 0-6 months: 3.00 (±0.23)	With obesity Full-mouth sites: 0-3 months: 0.30 (±0.06) 0-6 months: 0.54 (±0.07) Initially moderate sites (PD 4-6mm): 0-3 months: 0.70 (±0.13) 0-6 months: 1.10 (±0.15) Initially deep sites (PD ≥ 7mm): 0-3 months: 1.22 (±0.26) 0-6 months: 2.30 (±0.24)	*p*-value (<0.05) 0.08 0.04 0.31 0.16 0.07 0.04
Gonçalves et al. 2015 [32]	Total number of patients: 40 Non-obese: 20 Obese: 20 Age: Non-obese: 48.5 (±9.3) Obese: 50.0 (±4.5) p-value = 0.52 (n. sig.) Country: Brazil	n.r.	Non-obese: 3.4 (±0.6) – 2.9 (±0.6) *p*-value: sig. Obese: 3.6 (±0.6) – 3.3 (±0.5) *p*-value: sig.	Non-obese: 3.4 (±0.6) – 2.7 (±0.6) *p*-value: sig. Obese: 3.6 (±0.6) – 3.0 (±0.5) *p*-value: sig.	Non-obese: 3.4 (±0.6) – 2.6 (±0.6) *p*-value: sig. Obese: 3.6 (±0.6) – 2.9 (±0.4) *p*-value: sig.	Mean PPD values (± SD): After 3 months: Non-obese: 2.9 (±0.6) Obese: 3.3 (±0.5) *p* = 0.25 (n. sig.) After 6 months: Non-obese: 2.7 (±0.6) Obese: 3.0 (±0.5) *p* = 0.04 After 12 months: Non-obese: 2.6 (±0.6) Obese: 2.9 (±0.4) *p* = 0.03		
Lakkis et al. 2011 [33]	Total number of patients: 30 Surgery: 15 Control: 15 Age: Surgery: 45.6 (±11.1) Control: 48.5 (±12.0) p-value = 0.778 (n. sig.) Country: USA (Cleveland, Ohio)	4-6 weeks after therapy! Surgery: 2.6 (±0.6) – 2.2 (±0.5) *p*-value < 0.001 Control: 3.1 (±0.8) – 2.8 (±0.7) *p*-value < 0.001	n.r.	n.r.	n.r.	Surgery: 0.45 Control: 0.28 *p*-value = 0.007		
Suvan et al. 2014 [34]	Total number of patients: 260 Normal: 112 Overweight: 93 Obese: 55 Age: Normal: 46.30 (±8.38) Overweight: 47.27 (±8.93) Obese: 46.55 (±6.87) *p*-value = 0.701 (n. sig.) Country: Great Britain (London)	Summary of univariable GEE Analysis Results (Coefficient, 95 % Cl): BMI [kg/m^2^] to PPD at 2 months *p*-value = 0.055 Overweight to PPD at 2 months *p*-value = 0.372 (n. sig.) Obese to PPD at 2 months *p*-value = 0.064 (n. sig.) Summary of Multivariable GEE Analysis Results (Coefficient, 95 % Cl): BMI [kg/m^2^] to PPD at 2 months *p*-value = 0.013 Overweight to PPD at 2 months *p*-value = 0.63 (n. sig.) Obese to PPD at 2 months *p*-value = 0.031	n.r.	n.r.	n.r.	BMI and obesity were sig. associated with mean PPD after 2 months. (*p* = 0.055) Overweight was not sig. associated with PPD after 2 months (*p* = 0.372) BMI had a stat. sig. linear relationship with mean PPD after 2 months (*p* = 0.013). Overweight compared with normal BMI status did not demonstrate a stat. sig. association with mean PPD after 2 months (p = 0.63). Obesity compared with normal BMI status remained a stat. sig. predictor of mean PPD (*p* = 0.031) at 2 months in the same multivariable model.		

*CI* confidence interval, *CPNW* normal weight patients with periodontitis, *CPO* obese patients with periodontitis, *kg* kilogram, *m* meter, *mm* millimeter, *Nb ∆ PD* number of improving PD, *n.r.* not reported, *PD* probing depth, *PHNW* normal weight periodontally healthy control subjects, *PPD* periodontal pocket depth, *p* probability, *SD* standard deviation, *(n.) sig.* (not) significant, *stat.* statistically, *WHO* world health organisation

**Table 5:** Definition of periodontitis, periodontal assessment and definition of smoking

Author & year	Definition of chronic periodontitis	Periodontal assessment	Smokers
Al – Zahrani et al. 2012 [8]	≥ 20 teeth, CAL ≥ 3 mm at ≥ 30 of the sites (generalized moderate/severe chronic periodontits)	Full-mouth periodontal examination on all teeth excluding 3rd molars and partially erupted teeth	Smokers excluded
Bouaziz et al. 2015 [28]	≥ 12 teeth, ≥ 30 % of sites with CAL ≥ 3 mm (moderate-to-severe generalized chronic periodontitis)	Full-mouth clinical measurements included the following: 1) PI, 2) BOP, 3) PD (mm), 4) CAL (mm), PI, BOP, PD, and CAL measurements were performed using a manual periodontal probe at 6 sites per tooth, PD was defined as the distance from the free gingival margin to the bottom of the pocket/sulcus, and CAL was defined as the distance from the cemento-enamel junction to the bottom of the pocket/sulcus	Non-smoker or former smoker since ≥ 5 years
Duzagac et al. 2015 [29]	≥ 20 teeth, PD ≥ 4 mm in ≥ 30 % of periodontal sites, BOP in ≥ 50 % of periodontal sites, interproximal CAL > 2 mm in ≥ 20 % of periodontal sites, radiographic evidence of bone loss, as described by Armitage [177]	A Williams-type periodontal probe (UNC15, Hu-Friedy, Chicago, IL, USA) was used for the measurements of periodontal parameters, including PI (Silness–Löe) [178], GI (Löe–Silness) [179], BOP, PD and CAL, all clinical periodontal measurements were performed at 6 sites per tooth (mesiobuccal, mid-buccal, distobuccal, mesiolingual, mid-lingual and distolingual), excluding third molars	Smokers excluded
Eldin et al. 2013 [30]	n.r. (chronic periodontitis)	Full-mouth, 4 sites per tooth (mesio-buccal, mid-buccal, disto-buccal, mid-lingual) around each tooth	n.r.
Gonçalves et al. 2015 [31]	≥ 15 teeth excluding 3rd molars and teeth with advanced decay indicated for exodontia, >30 % of the sites with concomitant PD and CAL ≥4 mm and a minimum of 6 teeth distributed in the different quadrants presenting at least one site with PD and CAL ≥ 5 mm and BOP at baseline (generalized chronic periodontitis)	6 sites per tooth on all teeth excluding 3rd molars (distance between the gingival margin and the bottom of the sulcus/pocket [mm])	Smokers excluded
Gonçalves et al. 2015 [32]	≥ 15 teeth excluding 3rd molars and teeth with advanced decay indicated for exodontia, >30 % of the sites with concomitant PD and CAL ≥ 4 mm and a minimum of 6 teeth distributed in the different quadrants presenting at least one site with PD and CAL ≥ 5 mm and BOP at baseline (generalized chronic periodontitis)	6 sites per tooth (mesio-buccal, mid-buccal, disto-buccal, mesio-lingual, mid-lingual, disto-lingual) excluding third molars, with a manual periodontal probe (UNC15, Hu-Friedy, Chicago, IL, USA)	Current smokers excluded, smoking within the past 10 years excluded
Lakkis et al. 2011 [33]	≥ 20 teeth, mean CAL ≥ 2 mm (chronic periodontitis)	6 sites per tooth	n.r.
Suvan et al. 2014 [34]	PD > 5 mm and marginal alveolar bone loss > 30 % with > 50 % of the teeth affected (generalized severe periodontitis)	6 sites per tooth on all teeth present	Normal-group = 47 % smokers Overweight-group = 25 % smokers Obese-group = 15 % smokers *p* - value = 0.045 (sig. on *p* ≤ 0.05)

*BoP* bleeding on probing, *CAL* clinical attachment level, *GI* gingival index, *mm* millimeter, *n.r.* not reported, *p* probability, *PD* probing depth, *PI* plaque index, *sig.* significant

**Table 6:** Definition of obesity and systemic examination

Author & year	Definition of obesity	Systemic examination
Al – Zahrani et al. 2012 [8]	Normal-weight: BMI: 18.5 to < 25 kg/m^2^ Obese: BMI ≥ 30 kg/m^2^	Weight in kilograms and height in meters were measured; BMI: (weight/height^2^ [kg/m^2^])
Bouaziz et al. 2015 [28]	Normal-weight: BMI ≥ 18.5 kg/m^2^ and ≤ 25 kg/m^2^ Obese: BMI ≥ 30 kg/m^2^	Weight (kg), height (m), waist/hip circumferences (cm) weight was obtained using a calibrated scale,, height was measured using a measuring board, BMI: (weight/height^2^ [kg/m^2^]), waist measurements were taken at the level of the umbilicus (cm) hip measurements were taken at the greatest circumference with a measuring tape, WHR: ratio of waist to hip circumference
Duzagac et al. 2015 [29]	Normal-weight (CPNW): n.r. Obese (CPO): BMI ≥ 30 % and WHR > 0.95 for males and 0,80 for females	One trained examiner performed all anthropometric measurements: weight (kg), height (m), waist (cm), hip (cm), BMI: (weight/height^2^ [kg/m^2^]), WHR: ratio of waist to hip circumference, anthropometric measurements were repeated before the final follow-up visit to assess whether there was any change in BMI or WHR
Eldin et al. 2013 [30]	Overweight: BMI: 25-29.9 kg/m^2^ Obese: BMI ≥ 30 kg/m^2^ According to the WHO (3)	n.r.
Gonçalves et al. 2015 [31]	Without obesity: BMI 20-29.9 kg/m^2^ and WHR < 0.85 for females and < 0.9 for males With obesity: BMI ≥ 30 kg/m^2^ and < 40 kg/m^2^ and WHR ≥ 0.85 for females and WHR ≥ 0.9 for males	BMI: (weight/height^2^ [kg/m^2^]) WHR: ratio of waist to hip circumference
	Non-obese: BMI: 20-24.9 kg/m2 and WHR below that determined for obesity (i.e. WHR < 0.85 for women and WHR < 0.90 for men) (World Health Organization 2008) Obese: BMI ≥ 30 and < 40 kg/m2 and concomitant WHR ≥0.85 for women and WHR ≥ 0.90 for men.	One trained examiner performed all anthropometric measurements: weight (kg), height (m), waist (cm) and hip circumferences (cm) BMI: (weight/height^2^ [kg/m^2^]), WHR: ratio of waist to hip circumference, anthropometric measurements were reassessed at all follow-up visits to verify that the patients did not change their obese or non-obese status during the course of the study
Lakkis et al. 2011 [33]	Control: obese (BMI ≥ 30 kg/m^2^) Did not have the bariatric surgery nor did they lose weight to serve as a control group Surgery: obese (BMI ≥ 30 kg/m^2^) and ≥ 40 % loss of their excess weight at ≥ 6 months after bariatric surgery	BMI: (weight/height^2^ [kg/m^2^]) Percentage of weight loss after BS
Suvan et al. 2014 [34]	Normal: BMI: 18.5-24.99 kg/m^2^ Overweight: BMI: 25-29.99 kg/m^2^ Obese: BMI ≥ 30 kg/m^2^	BMI: (weight/height^2^ [kg/m^2^]) Based upon height and weight of the individual measured with a wall-mounted height measure and mechanical scales

*BF* body fat, *BMI* body mass index, *BS* bariatric surgery, *cm* centimeter, *CPNW* normal weight patients with periodontitis, *CPO* obese patients with periodontitis, *HDL* high-density lipoprotein, *kg* kilogram, *m* meter, *n. r.* not reported, *WC* waist circumference, *WHO* world health organization, *WHR* waist-hip-ratio

**Table 7:** Non-surgical periodontal treatment, treatment time and limitations

Author & Year	Non-surgical periodontal treatment	Treatment time; periodontal maintenance	Limitations
Al – Zahrani et al. 2012 [8]	Oral hygiene instructions, SRP performed by one operator	n.r.; n.r.	n.r.
Bouaziz et al. 2015 [28]	Oral hygiene instructions (brushing technique: Bass technique, use of interproximal hygiene devices, CHX mouthwash (0.12 %) twice a day for 7 days), SRP under local anesthesia using an ultrasonic device and manual curets, aim of each session was to remove biofilms and calculus of scaled roots, treatment protocol did not include antibiotic treatment, all treatments procedures were performed by the same periodontist (WB)	2-3 sessions for probing depth (PD) > 5 mm lasting 1 h each within 7 days; Oral hygiene was controlled at each appointment, hygiene instructions were repeated if needed at the 3-month examination, reinstruction of oral hygiene methods was performed if needed, and residual pockets ≥ 4 mm were systematically scaled and planed	During the study, two patients dropped out in each group for the following reasons: 1) two patients migrated; 2) one missed the 3-month examination; 3) one missed the final examination
Duzagac et al. 2015 [29]	Oral hygiene instructions, all kinds of dental treatments, such as fillings and adjustment of overhanging restorations, were completed immediately after patient enrollment, SRP using a reduced Gracey curette set (Gracey, SAS 5-6, SAS 7-8, SAS 11-12, SAS13- 14, Hu-Friedy) with an ultrasonic device (Dentsply/Cavitron, SPS, 30 K, TFI 1000, Dentsply, York, PA, USA) under local anesthesia, endpoint of treatment: a smooth tooth surface, patients received no adjunctive therapy (local or systemic antibiotics, NSAI, laser, ozonotherapy and mouth rinses), an experienced periodontist (EC) performed all treatment procedures (SRP, providing oral hygiene instructions and organizing patient recalls)	SRP in 4 sequential visits with 2 or 3 days intervals completed within 12 days, each appointment lasted 45 min; all patients received monthly periodontal maintenance, including oral hygiene reinforcement, scaling and polishing as necessary	8 were excluded for several reasons did not return for 3 months follow-up, pregnancy, weight loss
Eldin et al. 2013 [30]	Oral hygiene instructions, SRP	n.r.; periodontal maintenance (re-examination) performed weekly for 3 months after therapy	n.r.
Gonçalves et al. 2015 [31]	Oral hygiene instructions, Supragingival plaque and calculus removal, exodontia, filling overhang removal and provisional restoration as necessary, trained periodontist performed SRP with manual curets and ultrasonic device under local anesthesia, endpoint for each SRP appointment was “smoothness of the scaled roots.”	4 to 6 appointments lasting ≈ 1 h each, periodontal therapy was completed in 14 days; periodontal maintenance at 3 and 6 months after therapy	3 patients from the group without obesity and 6 from the group with obesity did not return for the 3-months follow-up visit and were excluded.
Author & Year	Non-surgical periodontal treatment	Treatment time; periodontal maintenance	Adverse events
Gonçalves et al. 2015 [32]	Oral hygiene instructions regarding brushing technique and use of dental floss, supragingival plaque and calculus removal, exodontia, provisional restoration and filling overhang removal, as necessary, trained periodontist performed SRP using manual curettes (Hu-Friedy, Chicago, IL, USA) and ultrasonic device (Cavitron Select SPC, Dentsply professional, York, PA, USA), under local anaesthesia, without use of local and/or systemic antimicrobials.	4 to 6 appointments lasting approximately 1 h each, Periodontal therapy completed within 2 weeks; All patients received periodontal maintenance every 3 months post-therapy	There were no patient and sampled site dropouts during the course of the study.
Lakkis et al. 2011 [33]	Oral hygiene instructions, SRP	n.r.; n.r.	n.r.
Suvan et al. 2014 [34]	Oral hygiene instructions, full-mouth mechanical periodontal debridement with hand and ultrasonic instruments performed with local anesthesia by the same clinician	Within a 24-h period of time; n.r.	No individuals in the sample had missing data for any of the covariates listed in this study.

*CHX* chlorhexidine, *min* minute, *mm* millimeter, *n.r.* not reported, *PD* pocket depth, *SRP* scaling and root planing

The quality of the included studies was assessed through the Newcastle-Ottawa Quality Assessment Scale (Table 8).

**Table 8:**
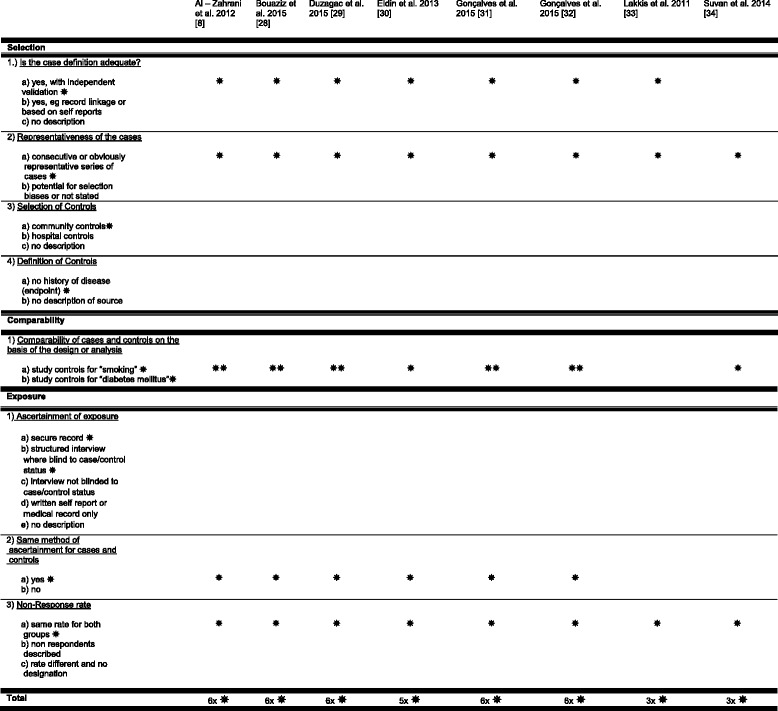
Newcastle – Ottawa Quality Assessment Scale

Stars represent “awards” for each quality item and serve as a quick visual assessment. A maximum of one ‘star’ for each item within the ‘Selection’/‘Exposure’ categories and a maximum of two ‘stars’ for ‘Comparability’ can be awarded

## Results

### Selection of studies

**Fig. 1:**
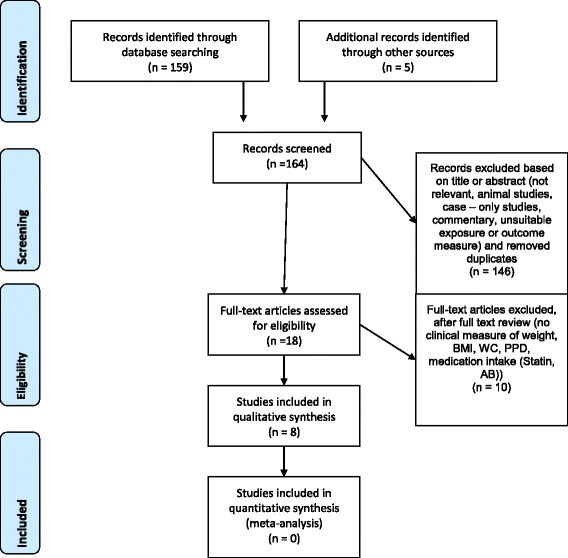
PRISMA 2009 flow diagram

Initially, 159 studies were identified by electronic search by the two reviewers (FAG, PRS). Full text analysis of the 18 potentially qualified reports led to exclusion of 10 other studies. Additional five titles [23–27] were identified by hand search but after full text analysis, all these articles had to be excluded based on the inclusion and exclusion criteria (Table 3). Therefore, eight publications [8, 28–34] from the electronic and hand search fulfilled the criteria. However, it was not possible to compare the raw data so that we had to reduce our analysis to a qualitative analysis. This process is summarized in a flow-chart (Fig. 1).

### Summary of studies: characteristics (PICO)

Total number of patients, demographic data, origin of study, outcome measurements 2, 3, 6 and 12 months after therapy and the impact of obesity on the treatment-outcome are summarized in Table 4.

Definition of chronic periodontitis, periodontal assessment and the amount of smokers included are depicted in Table 5.

The definition of obesity and systemic examination were summarized in Table 6. Non-surgical periodontal treatment, treatment time, periodontal maintenance and limitations are shown in Table 7.

### Population

Of the eight finally analyzed studies, clinical trials comprised 516 participants. One study [8] enrolled only women. The prevalence of smoking among male patients affected with periodontitis was so high that Al-Zahrani and co-workers [8] were unable to correct for smoking in the male population. Accordingly, only women were included in their study. Overall the studies comprised between 26 up to 260 subjects (Table 4).

The inclusion criteria “age” was defined in all but one study [30]. Three investigations [28, 33, 34] defined the age ≥18 years. The other studies defined a minimum age of 30 years [31, 32] or 35 years [8]. Duzagac et al. defined an age range from 25 to 55 years (Table 4).

Suvan et al. [34] included smokers into their study. Lakkis et al. [33] and Eldin et al.(30] did not mention the smoking status of the patients. All other studies [8, 28, 29, 31, 32] excluded smokers (Table 5).

Patients with diabetes, another important modifier of periodontal health or disease [35] were excluded in seven studies [8, 28–32, 34]. Lakkis et al. [33] did not report the presence or absence of diabetes.

### Intervention/Comparison

Each paper described the periodontal intervention as a non–surgical therapy. All studies [8, 28–34] applied scaling and root planing. Ultrasonic instruments and/or hand instruments were used in all studies (Table 7).

Two papers (8,34] reassessed the PPD 2 months after therapy. Two studies [29, 30] reassessed their patients after 3 months. Another two papers [28, 31] re-evaluated the patients after 3 and 6 months and one study [32] reassessed the PPD three, six and 12 months after therapy. Only Lakkis et al. [33] measured the periodontal pocket depths already 4 to 6 weeks after non-surgical therapy (Table 3). Oral hygiene was instructed additionally to the non–surgical periodontal therapy in all studies (Table 7).

Due to the heterogeneity of the study designs with respect to outcome measures and treatment protocol, as well as variation in study population, sample size, and/or statistical methods, a statistical synthesis of the results of the included studies was not possible. So the authors decided to analyze the papers on a qualitative way [22]. A meta-analysis was not possible.

### Outcome

Generally obese patients were found to have deeper periodontal pockets at baseline in all studies.

Three [8, 29, 30] of the eight papers [8, 28–34] reported no major negative impact of obesity on response to periodontal therapy in terms of PPD reduction (mm). Al-Zahrani et al. [8] assessed the reduction of PPD (mm) comparing obese with normal–weighted women. There was no statistically significant effect of obesity on treatment outcome. Duzagac et al. [29] assessed the clinical response to non-surgical periodontal treatment, according to the severity of periodontitis based on probing depth < 4 mm vs. ≥ 4 mm. Patients with and without obesity showed similar clinical healing in terms of percentage and number of sites with probing depth < 4 mm and ≥ 4 mm. So they failed to show an effect of obesity on the treatment outcome dependent on the severity of the disease. Eldin et al. [30] also found no effect when comparing an overweight group with an obese group. The difference between the groups in reduction of PPD was not significant (Table 4).

Five [28, 31–34] of the eight papers showed a negative effect of obesity on the healing after non-surgical periodontal therapy. Bouaziz et al. [28] revealed that normal-weight patients had a better response to periodontal treatment than obese patients. This effect was specially observed for moderate-to-deep pockets. This fact suggests that the more severe the periodontitis the more pronounced is the negative effect of obesity on periodontal treatment outcome. They showed in the multivariate analysis that obesity was significantly associated with percentage changes of PD > 5 mm and numbers of improving sites (p ≤ 0.05). In the univariate analysis all periodontal parameters improve more in patients suffering from more severe periodontitis at baseline. Other patient characteristics, like age, sex, obesity, and WHR, were not associated with periodontal parameter changes. Gonçalves et al. [31] showed that patients with obesity and chronic periodontitis had a lower PDD reduction than patients without obesity. The measurement of the reduction in PPD (mm) at full-mouth sites showed after 3 months a not statistically significant difference (*p* = 0.08) between the obese group compared to the group without obesity. However after 6 months there was a statistically significant difference (*p* = 0.04). At this time point, especially deep sites (PPD ≥ 7 mm) showed a significantly better result in the group without obesity (*p* = 0.04). Another study of Gonçalves et al. [32] reported that patients with obesity had a significantly greater mean PD (6 months *p*-value = 0.04, 12 months *p* value = 0.03) than patients without obesity at six and 12 months post-therapy. The data of a study by Suvan et al. [34] corroborated these findings and showed that obesity was an independent impact value of poorer periodontal treatment outcome 2 months after therapy. The extent of the association between poorer periodontal treatment and obesity was similar to that of smoking (*p* = 0.02). They worked with 260 patients. This secondary analysis consisted of individuals participating in five clinical studies of non-surgical periodontal therapy over a 7-year period. Lakkis et al. [33] selected 30 patients who were obese; 15 of them had previously undergone bariatric surgery, whereas the other half (*n* = 15) did not loose any weight and served as a control group. The bariatric surgery group reached a statistically significant greater mean PPD reduction (0.45 mm versus 0.28 mm) compared with the control (no surgery) group (*p* = 0.007; Table 4).

### Limitations

Some patients could not be reviewed or discontinued the study for personal reasons [28, 29, 31]. Weight loss and pregnancy were additional reasons to be excluded in the study of Duzagac et al. [29] (Table 7).

The quality of the included studies was evaluated through the Newcastle-Ottawa Quality Assessment Scale (Table 8).

## Discussion

This review focused on obesity and the outcome after non-surgical periodontal therapy and has shown that currently there is no really robust scientific evidence to reach solid conclusions and recommendations.

Three papers [8, 29, 30] were found, which did not find any statistically significant negative impact of obesity on the response to non-surgical periodontal therapy, whereas five papers [28, 31–34] showed the opposite, i.e. a clearly negative influence of obesity on the treatment outcomes.

With regard to the quality of evidence, seven included papers [8, 28, 29, 31–34] reported some limitations.

Al-Zahrani et al. [8] included only women. Gonçalves and co-workers [31] did not consistently apply the accepted definitions of overweight and obesity, but rather included the waist-to-hip-ratio (WHR) for their definitions, probably leading to inclusion of patients with a BMI inferior to 30 kg/m^2^ into the obesity group. Thus the results are difficult to interpret and compare with the other studies and are therefore not applicable for patients with a BMI ≥40 kg/m^2^ [31]. Nonetheless, these studies still show a clearly better tendency with regard to the treatment response for patients without obesity as defined in their study. Lakkis et al. [33] chose another interesting way to find an impact of obesity on the outcome of non-surgical periodontal treatment. They compared obese people who had undergone bariatric surgery (BS) with obese who did not. After weight loss in the BS group, a reduction in total adipocytes might have resulted in a decrease in adipokines and pro-inflammatory mediators released by those adipose cells. This systemic inflammatory reduction might have played a role in reducing the insulin resistance resulting in a better outcome after periodontal therapy as suggested by the authors [33]. Some limitations in the specific profile of the obese patients (nondiabetic, non-smoker) in the paper of Bouaziz et al. (28) may restrict the extrapolation of the results to the whole obese population. Furthermore, the small sample size may also limit the power of this study. Duzagac et al. [29] failed to include a control group of periodontally healthy controls with obesity. Additionally the mean periodontitis parameters were within the limits of “moderate” periodontitis, and the WHR and BMI values of these obese patients were predominantly below those characterizing morbid obesity. So, the results of this study may not be extrapolated to those with severe periodontitis or morbid obesity. The second included study of Gonçalves and co-workers [32] assumes that the high inter-patient variance in adipokine levels may reduce the statistical power to detect treatment effects, as previously reported. The results presented by Suvan et al. [34] may have been influenced by study limitations linked with unequal numbers in BMI categories and sample size. In addition, there may have been limitations with regard to the interpretation associated with the *post hoc* secondary analysis experimental design, although variation in clinical assessment and treatment was minimized by examiner and treatment clinician stability. This study did not constitute a higher level of evidence in the context of evidence-based health care levels of scientific evidence [34].

Overall, obesity is an obvious, visible stigma so that the studies cannot be considered blinded. This may be another possible bias in each of the studies.

Five studies [8, 28, 29, 31, 32] excluded smokers. Since smoking influences periodontal health these studies are biased [9] and may not be fully representative for the typical overall population. Overall it appears, however, that a positive effect of normal weight is present in non-smokers [28, 31, 32] and in smokers [34].

As mentioned before, the included studies differed in statistical methods, populations, sample sizes, definition of chronic periodontitis, definition of obesity, time of outcome measurement, smoking status, periodontal assessment and non-surgical periodontal therapy. Therefore, it was not only impossible to perform a meta-analysis but also draw clear conclusions.

Nonetheless there is a consensus in the studies that obesity is associated with different baseline PPD levels. The large cohort in the study of Suvan et al. [34] and the long term results of Gonçalves et al. [31, 32] may lead to the conclusion that obesity is an important negative factor which influences non-surgical periodontal therapy.

In summary, all studies [8, 28, 29, 31–34] included in this review validated the efficacy of non-surgical periodontal therapy, except the study of Eldin and co-workers [30] who did not report any efficacy of the therapy (Table 3). Clinically, it appears obvious that a therapy is necessary to reach periodontal health independent of the patient’s body mass index.

Because this systematic review provided only moderate evidence that obesity is an important factor for non-surgical periodontal therapy, future prospective cohort studies are needed to confirm these findings [36]. Such trials should be of high methodological quality. They should control important confounding factors such as smoking status, severity of chronic periodontitis, severity of obesity. Every patient should get the same periodontal treatment and periodontal maintenance. Overall, there is a possibility to solve the research question even though blinding of the examiners to obesity or non-obesity status is not practically possible.

Clinicians should know that obesity may have some influence on periodontal status and are likely to have a negative impact on the clinical outcome of conservative treatment, even if this systematic review found only five [28, 31–34] out of eight papers [8, 28–34] corroborating the influence of obesity on the clinical periodontal outcome focusing on PPD as surrogate parameter for periodontal healing.

Clinicians might consider a weight reduction diet as an additional treatment for periodontal health with a positive effect expected after 6 and 12 months [31, 32]. Also it should not be neglected that weight control has substantial other beneficial health effects which on their own justify such a recommendation.

## Conclusion

This systematic review indicates a possible negative relationship between obesity and poorer treatment outcome in obese patients after non-surgical therapy based on the results of five out of eight studies. Three of these studies denied an impact of obesity on the treatment. The potentially inferior healing response could be based on pathophysiological inflammatory models.

Baseline levels showed also a poorer periodontal health in patients with obesity compared with non-obese patients.

No study found any better dental health parameters in obese than in non-obese individuals, and although dental health may not be the most important target for arriving at a near normal body weight, a person who can keep his or her body weight near normal might, in addition to all other established health benefits, count on having better periodontal health than if they are obese.
